# 

**Published:** 1886-11

**Authors:** 


### COMMUNICATIONS.

Zymosis........ 531
Dental Education........ 537

### PROCEEDINGS.

Southern Dental Association........ 540

### SELECTIONS.

Chancre of the Gum........ 573
Purity of Drinking Water........ 575
Drunkard's Epilepsy........ 575
SALMAGUNDI........ 576

### EDITORIAL.

Ohio State Dental Society........ 579
Bibliographical........ 580
Biographical........ 582

